# Effects of self-acupressure on the fatigue-sleep disturbance-depression symptom cluster in breast cancer survivors: a phase III randomised controlled trial

**DOI:** 10.1007/s00520-026-11155-2

**Published:** 2026-09-17

**Authors:** Mengyuan Li, Wai Hang Kwok, Tao Wang, Renli Deng, Haiying Wang, Liqun Yao, Linda Deravin, Li Yang, Xiaojing Tian, Jing-Yu Benjamin Tan

**Affiliations:** 1 School of Nursing and Midwifery, University of Southern Queensland, Ipswich, QLD Australia; 2 Faculty of Health, Charles Darwin University, Brisbane, QLD Australia; 3 School of Nursing and Midwifery, Edith Cowan University, Joondalup, WA Australia; 4 Centre for Health Research, University of Southern Queensland, Springfield, QLD Australia; 5 The Affiliated Hospital of Zunyi Medical University, Zunyi, Guizhou China; 6 College of Nursing, Fujian University of Traditional Chinese Medicine, Fuzhou, Fujian China; 7 Office of the Pro Vice-Chancellor (First Nations), University of Southern Queensland, Ipswich, Australia; 8 The Second Affiliated Hospital of Zunyi Medical University, Zunyi, Guizhou China; 9 Guizhou Nursing Vocational College, Guiyang, Guizhou China; 10 School of Nursing and Midwifery, The University of Notre Dame Australia, Fremantle, WA Australia

**Keywords:** Self-acupressure, Breast neoplasm, Fatigue, Sleep disorder, Depression, Symptom cluster, Randomised controlled trial

## Abstract

**Purpose:**

To explore the effectiveness and safety of self-acupressure intervention on the fatigue-sleep disturbance-depression symptom cluster (FSDSC) among breast cancer (BC) survivors and to explore within-trial costs associated with the intervention.

**Methods:**

Eligible participants were randomly allocated to a true self-acupressure group, a sham self-acupressure group or a usual care group (36 in each). The primary outcomes were the FSDSC at both the individual symptom level and cluster symptom level. Individual symptoms were measured by the Brief Fatigue Inventory, the Pittsburgh Sleep Quality Index, and the Hospital Anxiety and Depression Scale-Depression, respectively. The cluster level was calculated as the mean of three separate 0-10 numeric rating scale scores for fatigue, depression, and sleep disturbance. The secondary clinical outcome was quality of life (QoL) measured by Functional Assessment of Cancer Therapy-Breast (FACT-B). Outcomes were assessed at baseline (T0, week 0), immediately after intervention (T1, week 7) and after follow-up (T2, week 19). Safety and related costs within trial were also reported.

**Results:**

Ninety-three participants completed the study. Significant group-by-time interactions were observed for all clinical outcomes and QoL. Subsequent pairwise comparisons showed that the true self-acupressure group had significantly better FSDSC than the other groups at T1 and T2 (all *p* < 0.01). At the individual symptom level, the true self-acupressure group also had significantly better PSQI and HADS-D scores than both comparison groups at T1 and T2 (all *p* < 0.05). For BFI, the true self-acupressure group had significantly lower scores than the usual care group at both T1 and T2 (both *p* < 0.0001), whereas the difference between the true and sham self-acupressure groups was only statistically significant at T2 (*p* = 0.0076). For QoL, the true self-acupressure group had significantly higher FACT-B total scores than the usual care group at T1 and T2 (both *p* < 0.0001). The difference in FACT-B total score between the true and sham self-acupressure groups was not statistically significant at T1 (*p* = 0.069) but was statistically significant at T2 (*p* = 0.0002). No adverse events were reported. During the trial, only one participant in each of the true self-acupressure and usual care groups reported healthcare resource use (medications plus clinical visits for symptom relief), with a sum cost of ¥931.06 and  ¥2247.44, respectively. The one-off cost of video production for the true self-acupressure group was ¥2879.

**Conclusions:**

The self-acupressure is a safe intervention that demonstrated positive effects in managing the FSDSC in BC survivors. While self-acupressure is recommended to support symptom management in cancer survivorship, its cost-effectiveness remains uncertain and warrants further investigation.

**Trial registration:**

ClinicalTrials.gov (NCT06412107), Date: 02/26/2024. The study protocol has been published.

**Supplementary information:**

The online version contains supplementary material available at https://doi.org/10.1007/s00520-026-11155-2.

## Introduction

With the advanced technologies in early detection and treatments, there has been considerable increase in the number of cancer survivors globally. There were over 53 million cancer survivors with 5-year survivorship worldwide in 2024 [1]. Among all cancer types, breast cancer (BC) in women has a relatively high 5-year survival rate of over 90% in high-income countries compared to over 80% in China [2, 3]. However, BC survivors often experience unpleasant symptoms during or after their cancer treatment which negatively affect their quality of life (QoL), particularly when these symptoms co-occur or interact biologically as a symptom cluster [4–6]. The fatigue-sleep disturbance-depression symptom cluster (FSDSC) is a common one among BC survivors [4, 7]. The occurrence of the FSDSC was mainly explained by the cytokine-induced sickness behaviour, of which pro-inflammatory cytokines trigger the fatigue, depression, and sleep disturbance [8, 9].

Effective management for the FSDSC in BC survivors is challenging. Guidelines from the Oncology Nursing Society, the National Comprehensive Cancer Network, and the American Society of Clinical Oncology emphasise the priority of non-pharmacological interventions in managing symptom clusters [10]. This is due to safety concerns (e.g. potential drug-drug interactions) and the absence of consistent evidence supporting pharmacological approaches [10]. Current evidence suggests that non-pharmacological interventions could be beneficial for managing the FSDSC in BC survivors [11, 12]. Cognitive behavioural therapy (CBT), acupressure, acupuncture, and physical exercise are proposed as effective and practical choices, since they may affect the physiological pathways associated with the FSDSC, significantly reducing the levels of pro-inflammatory cytokines [13–15] and thus potentially improving the FSDSC. However, the use of acupuncture may be constrained by potential risks such as soft tissue infection and subcutaneous ecchymosis given its invasive nature [16, 17]. Physical exercises such as dance or resistance exercise programmes are often energy-demanding, which may reduce participants’ interests and adherence, particularly among BC survivors experiencing persistent fatigue [18]. CBT typically requires multiple sessions with trained therapists, which may limit its accessibility in clinical settings [19]. Compared with those interventions, acupressure may offer a relatively safer and lower-cost option for cancer survivors, as it is non-invasive and can be self-administered following a short training without the need for ongoing clinical supervision [20].

Recent evidence has demonstrated potential benefits of acupressure in alleviating individual symptoms, such as fatigue, sleep issues, and depressive mood in cancer survivors (e.g. BC survivors) [20–23]. Benefits of acupressure have also been reported for other symptom clusters, such as the fatigue-pain-sleep disturbance-depression-anxiety symptom cluster and the pain-fatigue-sleep disturbance symptom cluster [11]. However, whether acupressure can effectively improve the FSDSC in BC survivors remains uncertain. Guided by the Medical Research Council Framework for Developing and Evaluating Complex Interventions [24, 25], a three-phase research programme was therefore proposed and conducted by our research team. In phase I, an evidence-based acupressure intervention protocol was developed and validated [26]. Somatic self-acupressure was chosen as the preferred modality as it allows cancer survivors to practise independently anytime and anywhere after minimal training [20]. In phase II, a pilot randomised controlled trial (RCT) was conducted to assess the feasibility and acceptability of the developed self-acupressure intervention protocol [27, 28]. A sham control group was also included to distinguish the specific and non-specific effects of acupressure [27, 29]. The phase II RCT demonstrated that self-acupressure is a feasible and safe approach with promising specific effects on the FSDSC in BC survivors [27, 28]. Based on these findings and participants’ feedback, several refinements were made to further optimise the study protocol to facilitate the study implementation of phase III RCT [27]. The Brief Fatigue Inventory (BFI) was adopted instead of the Multidimensional Fatigue Inventory (MFI) for fatigue assessment, as the MFI had a high proportion of missing values and was perceived by participants as burdensome to complete. WeChat (the most popular social media platform in China) combined with telephone contacts, was chosen as a follow-up strategy to improve participants’ adherence to home-based self-acupressure [30]. A 12-week follow-up period in the phase III RCT was implemented to capture the sustainability of self-acupressure’s beneficial effects. Moreover, a within-trial economic evaluation was also incorporated to identify the scope of costs and benefits, which could contribute to answering questions that matter most to decision makers. Given that self-acupressure showed significant positive effects on the FSDSC but not on QoL in the phase II RCT, the current study was designed as a subsequent fully powered RCT to further validate these findings. The study hypothesised that self-acupressure would alleviate the severity of the FSDSC at both cluster and single symptom levels and improve the QOL in BC survivors.

## Methods

### Study design and study settings

This phase III trial was a partially blinded, three-arm, sham-controlled RCT, consistent with the design of the phase II RCT. Participants were recruited from two tertiary hospitals affiliated with Zunyi Medical University. This trial has been registered at Clinicaltrials.gov (NCT06412107). A detailed study protocol was published separately [31], and the study methods were briefly outlined as follows.

### Participants, randomisation, and masking

Eligible participants were those who (1) were diagnosed with early-stage female BC without distant metastases (from stages I to IIIa); (2) had completed chemotherapy for at least 1 month and up to 3 years (to capture persistent symptoms); (3) had experienced at least moderate symptom cluster with a score of 4 or above on an NRS scale (from 0 = “no” to 10 = “worst”) for fatigue, sleep disturbance, and depression in the past month; (4) had no scheduled chemotherapy or radiotherapy during the study period; and (5) consented to participate in the study [31]. Participants were ineligible if they (1) were currently taking any medications to treat fatigue, sleep disturbance, and depression; (2) had received acupressure treatment within the past 6 months; (3) had severe weakness and/or cognitive impairment that prevented them from following study procedures; and (4) involved in other studies that might potentially affect this study’s outcomes [31].

The adherence of participants to the intervention protocol in phase II RCT was suboptimal, which has confounded the observed effect size [27]. Therefore, a prior sample size for the current study was calculated based on effect sizes reported in similar studies (ranging from 0.32 to 0.96) [32–34]. An effect size of 0.32 was adopted in the G*Power calculation based on a conservative consideration. Considering an anticipated 25% attrition rate during the intervention and follow-up periods [31], a final sample size of 108 patients (36 per group) was determined.

The randomisation sequences were generated by an independent randomisation person who was involved only in the randomisation procedure, using Sealed Envelope (https://www.sealedenvelope.com/). After the eligibility was established and the baseline assessment was completed, the study investigators contacted the person to identify group allocation. Participants were randomly assigned to one of the three groups with a block size of nine (1:1:1 allocation). Due to the nature of self-acupressure intervention, blinding for the participants in the usual care group was deemed impossible, while participants in the true and sham groups would not know the self-acupressure intervention was a true or sham method. A partially blinded outcome assessment for the true and sham self-acupressure groups was also maintained as the measures in this study were self-rated. Data were analysed by an independent statistician who was blinded to group allocation. A total of 93 participants completed the study: true self-acupressure group (*n* = 31), sham self-acupressure group (*n* = 31), and usual care group (*n* = 31).

### Intervention and procedures

Study intervention and procedures were consistent with the phase II RCT and were detailed in the published protocol [31]. All participants received an evidence-based and updated education booklet [35–37] with general strategies on the management of the FSDSC and usual care. Participants in the true self-acupressure group were required to perform acupressure at eleven acupoints (seven of which were bilateral) after training. The 11 acupoints were selected based on their relevance to alleviating fatigue, sleep disturbance, and depressive mood, including Zusanli (ST36), Sanyinjiao (SP6), Taixi (KI3), Hegu (LI4), Neiguan (PC6), Shenmen (HT7), Baihui (GV20), Qihai (CV6), Guanyuan (CV4), Yintang (EX-HN3), and Taichong (LR3). Participants in the sham self-acupressure group performed light pressure at eleven non-acupoints (seven of which were bilateral) after training. The non-acupoints were located 1–3 cm away from the acupoints used in the true self-acupressure group and were not on the meridians [26]. In addition, illustrations of the acupoint locations for the true self-acupressure and sham self-acupressure groups are provided in the supplementary figures. Both groups followed the same regimen: a 7-week period consisting of daily 36-min sessions at their convenient time, with each point stimulated for 2 min [26].

Clinical nurses at the study sites introduced this study to participants attending the hospitals for regular follow-up visits. Participants who were interested in study participation were referred to the study investigators for further eligibility assessment. Eligible participants who agreed to participate were asked to sign an informed consent and baseline assessment before group allocation. Participants assigned to either the true or sham self-acupressure group were required to attend face-to-face acupressure training sessions provided by the investigators [31]. Following the training, participants were asked to complete a return demonstration to ensure that they could perform self-acupressure correctly. The training session took about 20 min. Additional adhesive-coloured dots were applied to the acupoints to reinforce the correct locations on the body, when necessary. To further support participants’ self-acupressure at home, a home learning package with demonstration videos and charts was provided. During the 7-week intervention, daily WeChat reminders and weekly follow-ups were provided by an investigator to collect information about self-practice and any potential adverse events and to address any concerns related to self-practice at home [31].

### Outcome measures

The primary endpoints were changes in the FSDSC at both the single symptom level and cluster symptom level. The secondary outcomes were QoL, safety, and relevant economic outcomes within the trial. Clinical outcomes were collected using paper-based patient-reported questionnaires at baseline (T0), the end of intervention (T1, week 7), and the follow-up (T2, week 19). The primary and secondary outcomes were specified in the published study protocol paper [31] and briefly outlined below.

#### Demographic and clinical information questionnaire

A questionnaire was used to collect participants’ socio-demographic data (e.g. age, occupation, educational background) and medical information (e.g. cancer stage, medical history).

#### The FSDSC composite score: three separated numerical rating scale (NRS)

The 0–10 NRS is a widely used tool for measuring symptom intensity and has been successfully used to assess the severity and impact of various symptoms [38]. Its straightforward format makes it easily accessible to population with different educational backgrounds. Thus, symptom severity at the cluster level (the FSDSC composite score) was calculated as the average score of three single symptom ratings on “0 = no present” and “10 = as bad as you can imagine” [39, 40].

#### Brief Fatigue Inventory (BFI; 9 items)

The single fatigue symptom was assessed using the BFI Chinese version [41]. Each item is scored from 0 to 10. The score of fatigue is the mean value of the nine items. A high score represents a high level of fatigue. The Cronbach’s alpha value was found to be 0.92 for the severity section and 0.90 for the interference section [41].

#### The Pittsburgh Sleep Quality Index (PSQI; 19 items)

The single sleep disturbance symptom was assessed using the PSQI Chinese version [42]. The total score is from 0 to 21, with higher scores indicating a higher level of sleeping difficulty (Cronbach’s alpha = 0.79) [42].

#### Hospital Anxiety and Depression Scale-Depression (HADS-D; 7 items)

The single depression symptom was assessed using the HADS-D Chinese version [43]. Each item is scored from 0 to 3. A total score was calculated by summing seven items, yielding a range of 0 to 21. A high score represents a high level of depression (Cronbach’s alpha = 0.93) [43].

#### The Functional Assessment of Cancer Therapy-Breast (FACT-B; 37 items)

The Chinese version of the FACT-B with five subscales was used to assess QoL [44]. The Cronbach’s alpha values of the subscales ranged between 0.59 and 0.85, while the test-retest reliability values for all subscales ranged from 0.82 to 0.91 [44]. Each item is scored from 0 to 4. The total score (0–148) was obtained by summing all subscale scores. A higher score indicates a better QoL [44].

#### Safety

Any adverse events related to self-acupressure were collected through the regular contact between the participants and study investigators across the intervention period. Any discomfort or incidents were assessed and managed by the on-site professional acupuncture practitioner.

#### Measurement of costs

The costs for healthcare use and intervention-related expenses were collected as outlined in the protocol [31]. Costs related to healthcare use were collected prospectively and retrospectively from patient self-reports and medical records. A self-designed form was used to record healthcare utilisation costs related to fatigue, sleep disturbance and depression (e.g. general practice visit, medicine use). The intervention-related costs were tracked and recorded in detail throughout the study.

### Data analysis

Data analysis was performed generally in accordance with the published study protocol [31] with minor deviations from the original procedures. A brief description and clarification of the variations are provided in this section. Data cleaning, missing data handling, and baseline comparison were conducted in IBM SPSS Statistics 29.0.1.0. Baseline characteristics were summarised using descriptive statistics. Comparisons across the three study groups were conducted using one-way ANOVA or the Kruskal-Wallis *H* test for continuous variables, and the chi-square test or Fisher’s exact test for categorical variables, as appropriate [31]. Further analyses were conducted in R (version 4.3.3) using established packages for data wrangling, penalised regression, longitudinal modelling, and marginal-means estimation. Least absolute shrinkage and selection operator (LASSO) was used to select the potential covariates. The generalised estimating equations (GEE) model was conducted to identify the effects of group, time, group-by-time, and covariates on clinical outcomes. Estimated marginal means (EMMs) were obtained for each study group at each time point with covariate-adjusted outcomes. Pairwise contrasts among groups were computed within time and adjusted using Holm’s method.

The adjusted and unadjusted models yielded qualitatively similar estimates; however, the adjusted model provided more robust effect estimates by including potential covariates. This paper, therefore, primarily reported results from the adjusted model in the main analyses. Effect sizes were expressed as Cohen’s *d* (standardised mean differences) and interpreted using conventional small, medium, and large guidelines [45]. All statistical tests were two-sided with a significance threshold of 0.05. T2 outcome assessments were unavailable for 25 participants (7 in the true self-acupressure group, 9 in the sham self-acupressure group and 9 in the usual-care group) because data collection was temporarily interrupted during the PhD student’s transfer between universities. The GEE analyses used the available repeated outcome measurements for participants according to their originally assigned groups. For economic data, no discounting was necessary as the study period was less than 1 year. A cost-effectiveness and cost-utility analysis was not conducted due to the very limited data on healthcare resource use within trial. Moreover, clinical nurses assisting with participants’ recruitment and study investigators were provided as in-kind support in the study sites, and the only direct intervention-related cost was a one-off video production. The limited data collected did not allow for a statistically robust analysis; therefore, only descriptive reporting of intervention and healthcare resource costs is presented.

Some discrepancies between the study protocol and the actual implementation should be acknowledged. While the published protocol specified the use of SPSS for data analysis [31], both SPSS and R software were used to ensure more flexible and comprehensive statistical analysis. Least absolute shrinkage and selection operator (LASSO) with repeated cross-validation was used to estimate out-of-sample prediction error and to tally variable selection frequencies under the one-standard-error rule. The purpose was descriptive, mapping covariate importance to guide sensitivity analyses, rather than to hard-select predictors for the primary models. In addition, although the repeated-measures ANOVA was also initially planned, only the GEE was adopted to avoid over-analysis of the data. This variation was further justified by the superior robustness of GEE in handling missing data and its capacity to model within-subject correlations across repeated measurements.

## Results

### Participant recruitment and baseline characteristics

**Fig. 1:**
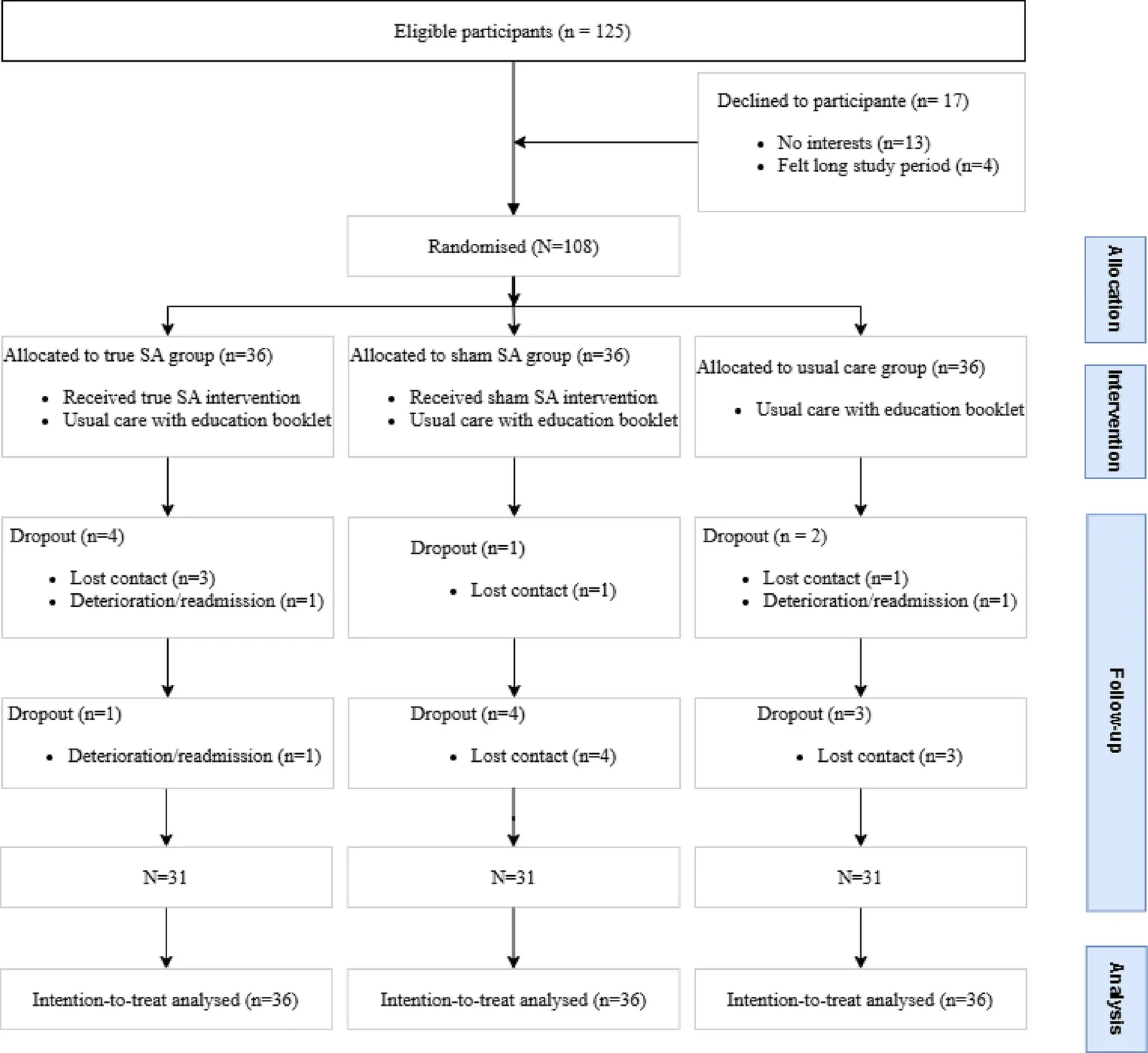
CONSORT flow diagram. Note: SA, self-acupressure

Participant recruitment was completed during an 8-month period (Fig. 1). A total of 108 BC survivors participated in the study, with 93 completing the study. The eligibility rate for the screened patients was 11.17% (125/1119), and the recruitment rate was 86.4% (108/125). The retention rate of the participants was 86.11% (93/108), and the attrition rate was 13.89% (15/108). The baseline characteristics of the participants (*n* = 108) are presented in Table 1. The enrolled participants were 48.63 ± 8.73 (mean ± SD) years old. Most of the participants (*n* = 84, 77.8%) were diagnosed with stage II BC. There were no statistically significant differences in socio-demographic or clinical characteristics between groups (*p* > 0.05).

**Table 1:** Participant characteristics at baseline

Variables	True SA	Sham SA	Usual care	Total	Statistics value	*p*
	*N* (%)	*N* (%)	*N* (%)			
Education status (*n* = 108)						
Primary school	10 (9.3)	16 (14.8)	16 (14.8)	42 (38.9)	5.872^a^	0.441
Middle school	12 (11.1)	12 (11.1)	14 (13.0)	38 (35.2)		
High school	7 (6.5)	4 (3.7)	2 (1.9)	13 (12.0)		
Tertiary education or above	7 (6.5)	4 (3.7)	4 (3.7)	15 (13.9)		
Religious background (*n* = 108)						
No	34 (31.5)	35 (32.4)	36 (33.3)	105 (97.2)	1.874^a^	0.771
Yes	2 (1.9)	1 (0.9)	0 (0.0)	3 (2.8)		
Ethnicity (*n* = 108)						
Han	33 (30.6)	34 (31.5)	33 (30.6)	100 (92.6)	8.255^a^	0.366
Miao	3 (2.8)	2 (1.9)	0 (0.0)	5 (4.6)		
Tujia	0 (0.0)	0 (0.0)	1 (0.9)	1 (0.9)		
Mongolian	0 (0.0)	0 (0.0)	1 (0.9)	1 (0.9)		
Others	0 (0.0)	0 (0.0)	1 (0.9)	1 (0.9)		
Marriage status (*n* = 108)						
Married	34 (31.5)	30 (27.8)	35 (32.4)	99 (91.7)	4.367^a^	0.141
Single	2 (1.9)	6 (5.6)	1 (0.9)	9 (8.3)		
Family income (monthly) (*n* = 107)						
≤ 3000 CNY	9 (8.4)	10 (9.3)	12 (11.2)	31 (29.0)	3.810^a^	0.717
3001–6000 CNY	15 (14.0)	16 (15.0)	11 (10.3)	42 (39.3)		
6001–10,000 CNY	7 (6.5)	9 (8.4)	10 (9.3)	26 (24.3)		
≥ 10,001 CNY	4 (3.7)	1 (0.9)	3 (2.8)	8 (7.5)		
Residence (*n* = 107)						
Rural/town	31 (29.0)	32 (29.9)	29 (27.1)	92 (86.0)	1.707^a^	0.450
City	5 (4.7)	3 (2.8)	7 (6.5)	15 (14.0)		
Living situation (*n* = 108)						
Living alone	1 (0.9)	11 (10.2)	6 (5.6)	18 (16.7)	12.588^b^	0.050
Living with children only	10 (9.3)	7 (6.5)	4 (3.7)	21 (19.4)		
Living with spouse only	17 (15.7)	12 (11.1)	18 (16.7)	47 (43.5)		
Living with whole family	8 (7.4)	6 (5.6)	8 (7.4)	22 (20.4)		
Primary caregiver (*n* = 108)						
None	9 (8.3)	15 (13.9)	11 (10.2)	35 (32.4)	5.733^a^	0.183
Family members	27 (25.0)	19 (17.6)	21 (20.4)	68 (63.0)		
Others	0 (0.0)	2 (1.9)	3 (2.8)	5 (4.6)		
Employment status (*n* = 108)						
Retired	5 (4.6)	5 (4.6)	1 (0.9)	11 (10.2)	3.628^a^	0.467
Employed	9 (8.3)	10 (9.3)	10 (9.3)	29 (26.9)		
Unemployed	22 (20.4)	21 (19.4)	25 (23.1)	68 (63.0)		
Medical insurance (*n* = 108)						
No	1 (0.9)	0 (0.0)	0 (0.0)	1 (0.9)	1.836^a^	1.000
Yes	35 (32.4)	36 (33.3)	36 (33.3)	107 (99.1)		
Medicare payment (*n* = 108)						
Fully self-paid (100%)	1 (0.9)	0 (0.0)	0 (0.0)	1 (0.9)	2.559^a^	0.738
Mostly self-paid (> 50% out-of-pocket)	20 (18.5)	19 (17.6)	17 (15.7)	56 (51.9)		
Partially self-paid (< 50% out-of-pocket)	15 (13.9)	17 (15.7)	19 (17.6)	51 (47.2)		
Fully reimbursed	0 (0.0)	0 (0.0)	0 (0.0)	0 (0.0)		
Cancer stage (*n* = 108)						
Stage I	5 (4.6)	3 (2.8)	6 (5.6)	14 (13.0)	4.845^a^	0.572
Stage IIA	17 (15.7)	24 (22.2)	16 (14.8)	57 (52.8)		
Stage IIB	10 (9.3)	6 (5.6)	11 (10.2)	27 (25.0)		
Stage IIIA	4 (3.7)	3 (2.8)	3 (2.8)	10 (9.3)		
Surgery type (*n* = 108)						
Modified radical mastectomy	17 (15.7)	17 (15.7)	17 (15.7)	51 (47.2)	2.191^a^	0.995
Breast cancer simple excision	10 (9.3)	9 (8.3)	11 (10.2)	30 (27.8)		
Breast-conserving surgery	9 (8.3)	9 (8.3)	8 (7.4)	26 (24.1)		
Others	0 (0.0)	1 (0.9)	0 (0.0)	1 (0.9)		
Chemotherapy regimen received (*n* = 108)						
AC/ACT	14 (13.0)	14 (13.0)	16 (14.8)	44 (40.7)	6.219^a^	0.634
EC/TEC	13 (12.0)	13 (12.0)	16 (14.8)	42 (38.9)		
TC/TAC	4 (3.7)	7 (6.5)	2 (1.9)	13 (12.0)		
Others	1 (0.9)	1 (0.9)	1 (0.9)	3 (2.8)		
Unclear	4 (3.7)	1 (0.9)	1 (0.9)	6 (5.6)		
Allergy history (*n* = 108)						
No	32 (29.6)	31 (28.7)	34 (31.5)	97 (89.8)	1.449^a^	0.617
Yes	4 (3.7)	5 (4.6)	2 (1.9)	11 (10.2)		
Completed chemotherapy cycles (*n* = 105)						
2–4 cycles	13 (12.4)	15 (14.3)	17 (16.2)	45 (42.9)	3.059^b^	0.555
5–6 cycles	10 (9.5)	9 (8.6)	12 (11.4)	31 (29.5)		
7–8 cycles	11 (10.5)	12 (11.4)	6 (5.7)	29 (27.6)		
Received targeted therapy (*n* = 106)						
No	29 (27.4)	27 (25.5)	31 (29.2)	87 (82.1)	0.992^b^	0.598
Yes	6 (5.7)	8 (7.5)	5 (4.7)	19 (17.9)		
Received radiotherapy (*n* = 105)						
No	20 (19.0)	25 (23.8)	15 (14.3)	60 (57.1)	5.833^b^	0.065
Yes	15 (14.3)	10 (9.5)	20 (19.0)	45 (42.9)		
Received hormone therapy (*n* = 106)						
No	4 (3.8)	3 (2.8)	1 (0.9)	8 (7.5)	2.011^a^	0.351
Yes	31 (29.2)	32 (30.2)	35 (33.0)	98 (92.5)		
Family history of BC (*n* = 107)						
No	34 (31.8)	34 (31.8)	35 (32.7)	103 (96.3)	0.668^a^	1.000
Yes	1 (0.9)	2 (1.9)	1 (0.9)	4 (3.7)		
Diabetes (*n* = 108)						
No	35 (32.4)	36 (33.3)	35 (32.4)	106 (98.1)	1.261^a^	1.000
Yes	1 (0.9)	0 (0.0)	1 (0.9)	2 (1.9)		
Hypertension (*n* = 108)						
No	34 (31.5)	33 (30.6)	32 (29.6)	99 (91.7)	0.784^a^	0.907
Yes	2 (1.9)	3 (2.8)	4 (3.7)	9 (8.3)		
FSDSC composite (mean/SD)	5.79 (1.04)	5.51 (0.87)	5.58 (0.93)	5.63 (0.95)	0.834^c^	0.437
BFI total (mean/SD)	4.89 (1.48)	4.56 (1.30)	4.61 (1.49)	4.69 (1.42)	0.559^c^	0.573
PSQI total (median/IQR)	12.00 (3)	13.00 (3)	12.50 (3)	12.50 (3)	0.487^d^	0.784
HADS-D (median/IQR)	10.00 (4)	10.00 (3)	9.00 (3)	10.00 (3)	1.058^d^	0.589
FACT-B total (mean/SD)	78.29 (16.37)	82.00 (15.34)	82.20 (15.78)	80.83 (15.79)	0.698^c^	0.500
Age (mean/SD)	48.25 (7.49)	47.61 (9.58)	50.03 (9.04)	48.63 (8.73)	0.738^c^	0.481

^a^Fisher’s exact test, ^b^chi-square test, ^c^ANOVA, ^d^Kruskal-Wallis *H* test, *SA* self-acupressure; chemotherapy regimen, *A* adriamycin, *E* epirubicin, *C* cytoxan, *T* taxol or taxotere, *FACT-B* functional assessment of cancer therapy-breast, *PSQI* pittsburgh sleep quality index, *HADS-D* hospital anxiety and depression scale-depression, *BFI* brief fatigue inventory, *HADS-A* hospital anxiety and depression scale-anxiety, *BC* breast cancer

### Participants’ adherence to the self-acupressure intervention

Most participants in the true (91.7%) and sham self-acupressure groups (75%) performed more than 30 min daily. During the entire study period, approximately one-third of participants in the true self-acupressure group (27.8%) and less than one-sixth in the sham self-acupressure group (13.9%) completed the practice for 49 days. No significant difference in daily session (*p* = 0.151) and total duration (*p* = 0.143) was found between groups.

### Effects of self-acupressure on the FSDSC

**Fig. 2:**
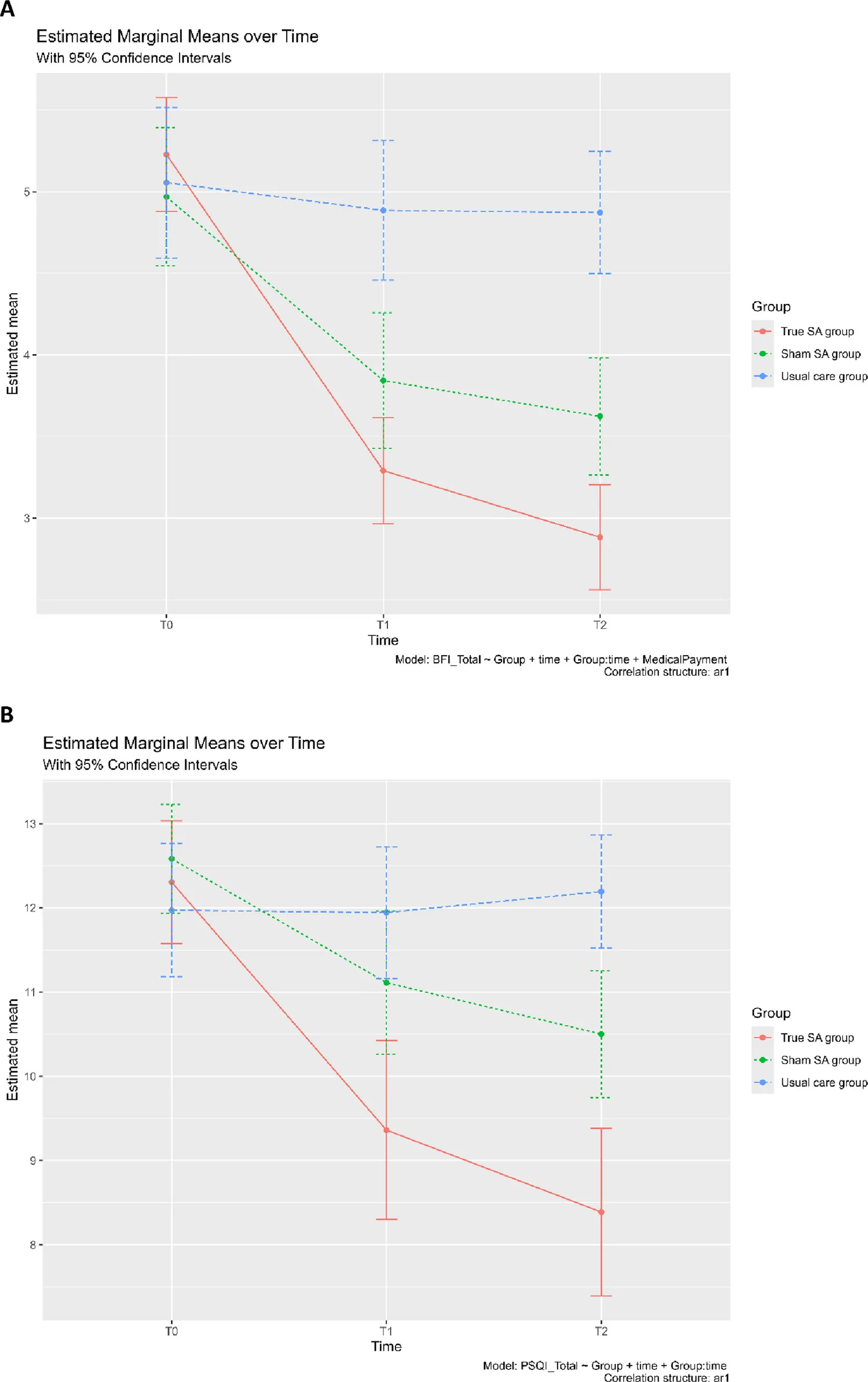

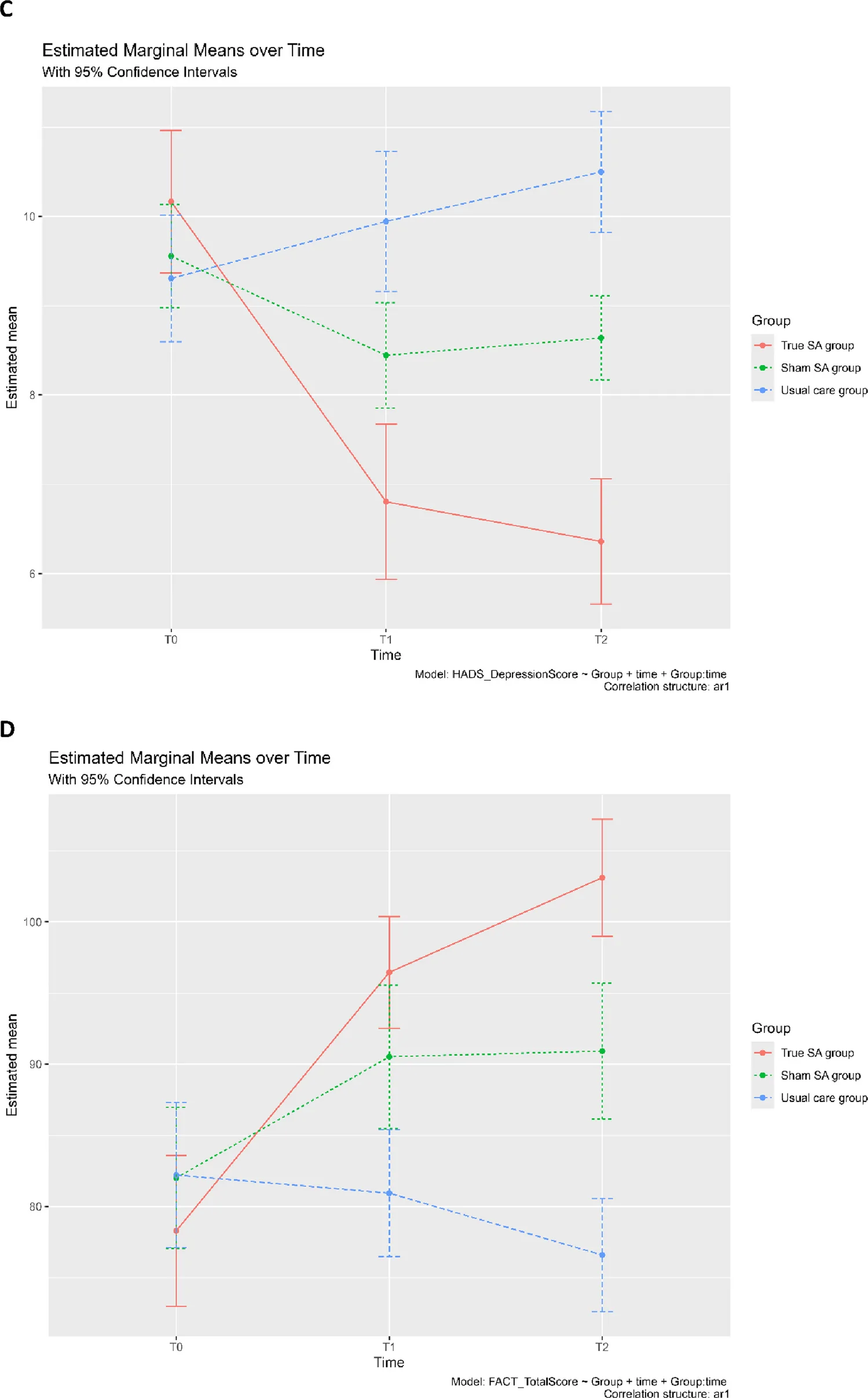

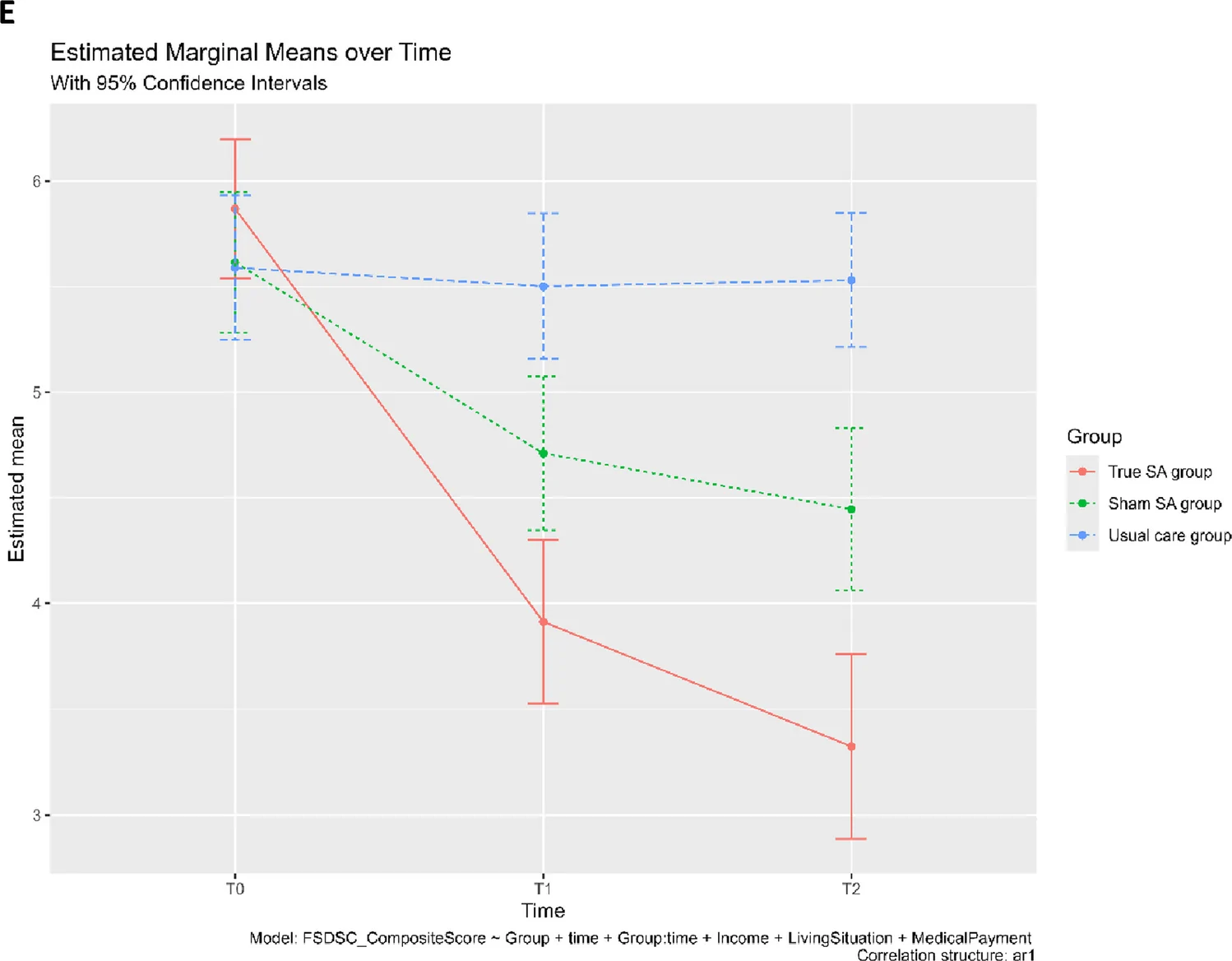
Changes in cluster level and individual symptom level of the fatigue-sleep disturbance-depression symptom cluster, and quality of life in the three study groups. Covariate-adjusted estimated marginal means (± 95% CI) at T0, T1, T2 for: **A** Brief Fatigue Inventory (BFI) total score, **B** Pittsburgh Sleep Quality Index (PSQI) total score, **C** Hospital Anxiety and Depression Scale-Depression (HADS-D) score, **D** Functional Assessment of Cancer Therapy-Breast (FACT-B) total score, **E** The fatigue-sleep disturbance-depression (FSDSC) composite score. Colours: true self-acupressure group (red line), sham self-acupressure group (green line), usual care (blue line)

The adjusted GEE analysis showed significant group-by-time interactions for the FSDSC scores, indicating greater reductions in the true self-acupressure group compared with the sham and usual care groups over time. Compared with the usual care group and T0, the true self-acupressure group demonstrated significantly greater reductions in FSDSC scores at T1 (*B* = 1.87, *p* < 0.001) and T2 (*B* = 2.49, *p* < 0.001) (Table 3). Similarly, significant reductions were observed at T1 (*B* = 1.05, *p* < 0.001) and T2 (*B* = 1.38, *p* < 0.001) when compared with the sham self-acupressure group and T0 in the adjusted model (Table 3). A confounding effect was observed with “Medical payment (Partially self-paid)” (*p* = 0.037) in the model (Table 3). Between-group comparisons also indicated that the FSDSC composite score in the true self-acupressure was significantly lower than those in the usual care group and sham self-acupressure group with large effect sizes at T1 (all *p* < 0.001) and T2 (all *p* < 0.001) (Table 2 and Fig. 2). The sham self-acupressure group also significantly outperformed the usual care group at T1 (*p* < 0.001) and T2 (*p* < 0.001). The true self-acupressure group showed a large effect compared with the usual care group at T1 (− 1.68) and a stronger effect at T2 (− 2.33), while effect sizes between the sham self-acupressure group and the usual care group at T1 and T2 were − 0.84 and −1.15. Moreover, the effective sizes between true self-acupressure and sham self-acupressure groups at T1 and T2 were −0.84 and −1.19 (Table 2).

**Table 2:** Estimated marginal means from adjusted GEE and standardised effect sizes for pairwise contrasts (using a first-order autoregressive correlation structure)

Outcomes	Time point	True SA group (T)	Sham SA group (S)	Usual care group (U)	T vs S		T vs U		S vs U	
		EMM (95% CI)	EMM (95% CI)	EMM (95% CI)	*p*	Effect size	*p*	Effect size	*p*	Effect size
FSDSC	T0	5.87 (5.54, 6.20)	5.61 (5.28, 5.95)	5.59 (5.25, 5.93)	0.4863	0.27	0.486	0.29	0.8977	0.03
	T1	3.91 (3.53, 4.30)	4.71 (4.35, 5.08)	5.50 (5.16, 5.85)	**0.0015**	−0.84	** < 0.0001**	−1.68	**0.0005**	−0.84
	T2	3.32 (2.89, 3.76)	4.45 (4.06, 4.83)	5.53 (5.21,5.85)	** < 0.0001**	−1.19	** < 0.0001**	−2.33	** < 0.0001**	−1.15
BFI	T0	5.23 (4.88,5.58)	4.97 (4.55,5.39)	5.06 (4.59, 5.52)	1.0000	0.18	1.0000	0.12	1.0000	−0.06
	T1	3.29 (2.97,3.61)	3.84 (3.43, 4.26)	4.89 (4.46, 5.31)	0.0634	−0.39	** < 0.0001**	−1.12	**0.0007**	−0.73
	T2	2.88 (2.56, 3.20)	3.62 (3.26, 3.98)	4.87 (4.50, 5.25)	**0.0076**	−0.52	** < 0.0001**	−1.40	** < 0.0001**	−0.88
PSQI	T0	12.31 (11.58, 13.03)	12.58 (11.93,13.23)	11.97 (11.18, 12.76)	1.0000	−0.12	1.0000	0.15	0.7210	0.27
	T1	9.36 (8.30, 10.42)	11.11 (10.26, 11.96)	11.94 (11.16, 12.72)	**0.0238**	−0.78	**0.0004**	−1.16	0.1562	−0.37
	T2	8.39 (7.40, 9.38)	10.50 (9.74, 11.26)	12.19 (11.52, 12.87)	**0.0020**	−0.94	** < 0.0001**	−1.70	**0.0020**	−0.76
HADS-D	T0	10.17 (9.37, 10.96)	9.56 (8.98, 10.13)	9.31 (8.60, 10.01)	0.4449	0.28	0.3392	0.40	0.5901	0.12
	T1	6.81 (5.94, 7.67)	8.44 (7.85, 9.04)	9.94 (9.16, 10.73)	**0.0046**	−0.75	** < 0.0001**	−1.44	**0.0046**	−0.69
	T2	6.36 (5.66, 7.06)	8.64 (8.17, 9.11)	10.50 (9.82, 11.18)	** < 0.0001**	−1.05	** < 0.0001**	−1.90	** < 0.0001**	−0.86
FACT-B total	T0	78.29 (72.99, 83.58)	82.00 (77.04, 86.96)	82.20 (77.10, 87.31)	0.8860	−0.24	0.8860	−0.25	0.9556	−0.01
	T1	96.45 (92.53, 100.38)	90.52 (85.47, 95.57)	80.93 (76.47, 85.39)	0.0689	0.38	** < 0.0001**	0.98	**0.0109**	0.61
	T2	103.12 (98.99, 107.25)	90.91 (86.13, 95.69)	76.59 (72.61, 80.56)	**0.0002**	0.77	** < 0.0001**	1.68	** < 0.0001**	0.91
PWB subscale	T0	19.31 (18.12, 20.49)	19.73 (18.34, 21.12)	20.19 (19.15, 21.24)	1.0000	−0.11	0.8054	−0.24	1.0000	−0.12
	T1	23.03 (22.03, 24.03)	21.39 (20.09, 22.69)	20.25 (19.18, 21.31)	0.1011	0.44	**0.0007**	0.74	0.1828	0.31
	T2	23.83 (22.89, 24.78)	21.58 (20.50, 22.67)	18.69 (17.77, 19.62)	**0.0022**	0.60	** < 0.0001**	1.37	**0.0002**	0.77
SWB subscale	T0	13.12 (11.11, 15.13)	13.69 (12.04, 15.33)	13.60 (11.51, 15.68)	1.0000	−0.10	1.0000	−0.08	1.0000	0.02
	T1	14.76 (13.39, 16.13)	14.46 (12.96, 15.96)	13.20 (11.43, 14.98)	0.7741	0.05	0.5183	0.26	0.5731	0.21
	T2	16.56 (15.38, 17.75)	14.50 (12.99, 16.00)	13.11 (11.25, 14.96)	0.0680	0.35	**0.0065**	0.59	0.2530	0.24
EWB subscale	T0	14.98 (13.60, 16.36)	14.99 (13.30, 16.69)	15.17 (13.66, 16.69)	1.0000	0.00	1.0000	−0.04	1.0000	−0.04
	T1	18.70 (17.42,19.98)	17.27 (15.71, 18.83)	14.62 (13.31, 15.92)	0.1198	0.31	** < 0.0001**	0.90	**0.0136**	0.58
	T2	19.67 (18.44, 20.90)	16.94 (15.52, 18.36)	13.54 (12.29, 14.78)	**0.0012**	0.60	** < 0.0001**	1.35	**0.0002**	0.75
FWB subscale	T0	8.81 (7.48, 10.13)	10.22 (8.73, 11.71)	10.73 (9.29, 12.16)	0.3265	−0.32	0.1622	−0.43	0.6311	−0.11
	T1	13.14 (11.82, 14.46)	12.19 (10.72, 13.67)	9.67 (8.58, 10.76)	0.3490	0.21	**0.0003**	0.78	**0.0141**	0.57
	T2	14.81 (13.46, 16.15)	12.25 (10.83,13.67)	9.08 (8.14, 10.02)	**0.0104**	0.58	** < 0.0001**	1.29	**0.0006**	0.72
BCS subscale	T0	23.64 (22.07, 25.21)	24.53 (23.25, 25.81)	23.88 (22.66, 25.10)	1.0000	−0.21	1.0000	−0.06	1.0000	0.16
	T1	28.39 (27.38, 29.39)	26.36 (25.00, 27.72)	24.57 (23.32, 25.81)	**0.0375**	0.48	** < 0.0001**	0.91	0.0563	0.43
	T2	29.81 (29.01, 30.60)	26.81 (25.47, 28.14)	23.54 (22.43, 24.64)	**0.0003**	0.71	** < 0.0001**	1.49	**0.0003**	0.78

*EMM* estimated mean, *SA* self-acupressure, *95% CI* 95% confidence interval, *GEE* generalised estimating equations, *T0* baseline, *T1* post-intervention, *T2* post-follow-up, *FACT-B* the functional assessment of cancer therapy – breast, *PSQI* the pittsburgh sleep quality index, *HADS-D* hospital anxiety and depression scale – depression, *BFI* brief fatigue inventory, *FSDSC* fatigue-sleep disturbance-depression symptom cluster, *PWB* physical well-being, *SWB* social well-being, *EWB* emotional well-being, *FWB* functional well-being, *BCS* breast cancer subscale, Bold entries indicate statistical significance at *p < *0.05

Model specification: FSDSC composite score: group, time, group by time, income, living situation, medical payment

BFI score: group, time, group by time, medical payment

PSQI total score: group, time, group by time

HADS-D Score: group, time, group by time

FACT total score: group, time, group by time

PWB subscale: group, time, group by time

SWB subscale: group, time, group by time

EWB subscale: group, time, group by time, medical payment, allergy history, primary caregiver

FWB subscale: group, time, group by time

BCS subscale: group, time, group by time

### Effects of self-acupressure on fatigue

The adjusted GEE model indicated that the true self-acupressure group achieved significant improvements in fatigue (assessed by BFI). Compared with the usual care group and T0, the BFI total scores were significantly lower in the true self-acupressure group at T1 (*B* = 1.77, *p* < 0.001) and T2 (*B* = 2.16, *p* < 0.001) in the adjusted GEE model (Table 3). Similar significant improvements were also indicated at T1 (*B* = 0.81, *p* < 0.005) and T2 (*B* = 1.0, *p* < 0.001) when compared with the sham self-acupressure group and T0 in the adjusted model (Table 3). Between-group comparisons at T1 and T2 found significant differences in the BFI scores between the true self-acupressure and usual care group, with large effect sizes (Table 2 and Fig. 2). The sham self-acupressure group also outperformed significantly usual care group at both T1 and T2 (all *p* < 0.001, Table 2). However, between-group comparisons only indicated a significantly lower BFI total score at T2 (effect size = −0.52) between true self-acupressure group and sham self-acupressure group.

**Table 3:** Adjusted GEE model for BFI, PSQI, HADS-D, and the FSDSC composite scores (using a first-order autoregressive correlation structure)

Predictor term	Coefficient estimate	Standard error	Wald statistic	*p*-value
Dependent variable: BFI total score				
Intercept	6.08	0.12	2374.81	< 0.0001
Group = sham SA (vs true SA)	−0.26	0.31	0.72	0.3951
Group = usual care (vs true SA)	−0.17	0.32	0.28	0.5943
Time = T1 (vs T0)	−1.94	0.23	73.96	** < 0.0001**
Time = T2 (vs T0)	−2.34	0.23	104.65	** < 0.0001**
Medical payment = mostly self-paid (> 50%)	−0.89	0.21	18.96	** < 0.0001**
Medical payment = partially self-paid (< 50%)	−1.68	0.23	54.32	** < 0.0001**
Interaction: sham SA × T1	0.81	0.29	8.08	**0.0045**
Interaction: usual care × T1	1.77	0.25	48.33	** < 0.0001**
Interaction: sham SA × T2	1.00	0.29	12.15	**0.0005**
Interaction: usual care × T2	2.16	0.26	69.22	** < 0.0001**
Dependent variable: PSQI total score				
Intercept	12.31	0.37	1104.76	** < 0.0001**
Group = sham SA (vs true SA)	0.28	0.50	0.31	0.5752
Group = usual care (vs true SA)	−0.33	0.55	0.37	0.5417
Time = T1 (vs T0)	−2.94	0.50	34.27	** < 0.0001**
Time = T2 (vs T0)	−3.92	0.46	73.98	** < 0.0001**
Interaction: sham SA × T1	1.47	0.60	6.04	**0.0140**
Interaction: usual care × T1	2.92	0.57	26.45	** < 0.0001**
Interaction: sham SA × T2	1.83	0.56	10.74	**0.0010**
Interaction: usual care × T2	4.14	0.53	61.50	** < 0.0001**
Dependent variable: HADS-D score				
Intercept	10.17	0.41	628.90	** < 0.0001**
Group = sham SA (vs true SA)	−0.61	0.50	1.49	0.2215
Group = usual care (vs true SA)	−0.86	0.54	2.52	0.1121
Time = T1 (vs T0)	−3.36	0.38	78.59	** < 0.0001**
Time = T2 (vs T0)	−3.81	0.38	100.03	** < 0.0001**
Interaction: sham SA × T1	2.25	0.49	20.90	** < 0.0001**
Interaction: usual care × T1	4.00	0.50	65.08	** < 0.0001**
Interaction: sham SA × T2	2.89	0.49	34.40	** < 0.0001**
Interaction: usual care × T2	5.00	0.52	92.23	** < 0.0001**
Dependent variable: FSDSC composite score				
Intercept	6.13	0.27	522.46	** < 0.0001**
Group = sham SA (vs true SA)	−0.25	0.21	1.47	0.2251
Group = usual care (vs true SA)	−0.28	0.20	1.96	0.1611
Time = T1 (vs T0)	−1.95	0.16	142.43	** < 0.0001**
Time = T2 (vs T0)	−2.54	0.21	143.53	** < 0.0001**
Income = 3001–6000 RMB	−0.43	0.22	3.83	0.0503
Income = 6001–10,000 RMB	0.16	0.24	0.43	0.5096
Income ≥ 10,001 RMB	−0.16	0.38	0.17	0.6808
Living situation = with children only	−0.23	0.30	0.60	0.4373
Living situation = with spouse only	0.26	0.26	1.02	0.3132
Living situation = with family (extended)	0.10	0.27	0.14	0.7099
Medical payment = mostly self-paid (> 50%)	−0.08	0.18	0.18	0.6688
Medical payment = partially self-paid (< 50%)	−0.48	0.23	4.37	0.0365
Interaction: sham SA × T1	1.05	0.19	31.20	** < 0.0001**
Interaction: usual care × T1	1.87	0.19	100.84	** < 0.0001**
Interaction: sham SA × T2	1.38	0.24	31.57	** < 0.0001**
Interaction: usual care × T2	2.49	0.24	111.66	** < 0.0001**

*T0* baseline, *T1* post-intervention, *T2* post-follow-up, *SA* self-acupressure, *GEE* generalised estimating equations, *BFI* brief fatigue inventory, *PSQI* the pittsburgh sleep quality index, *HADS-D* hospital anxiety and depression scale – depression, *FSDSC* fatigue-sleep disturbance-depression symptom cluster, *RMB* Chinese yuan; interaction means, Bold entries indicate statistical significance at *p < *0.05

### Effects of self-acupressure on sleep disturbance

The results of the adjusted GEE model identified that self-acupressure resulted in a greater relief of sleep disturbance (assessed by the PSQI). The PSQI scores were significantly lower in the true self-acupressure at T1 (*B* = 2.92, *p* < 0.001) and T2 (*B* = 4.14, *p* < 0.001) when compared with the usual care group and T0. When compared with the sham self-acupressure and T0, the true self-acupressure also performed better at T1 (*B* = 1.47, *p* = 0.014) and T2 (*B* = 1.83, *p* = 0.001). At T1 and T2, between-group comparisons indicated that the PSQI scores in the true self-acupressure group were significantly lower than those in sham self-acupressure and usual care groups (all *p* < 0.05), see Table 2 and Fig. 2. However, sham self-acupressure group outperformed usual care only at T2 (*p* = 0.002, Table 2). The true self-acupressure group showed large effects compared with usual care at both T1 (− 1.16) and T2 (− 1.70), while effect sizes between sham self-acupressure and usual care were smaller at T2 (− 0.76). In addition, the effect sizes between true self-acupressure and sham self-acupressure were − 0.78 at T1 and − 0.94 at T2, confirming stronger and sustained benefits of true self-acupressure over time (Table 2).

### Effects of self-acupressure on depression

The adjusted GEE results indicated that self-acupressure contributed to significant relief of depression (assessed by the HADS-D). The HADS-D scores were significantly lower in the true self-acupressure group at T1 (*B* = 4.00, *p* < 0.001) and T2 (*B* = 5.00, *p* < 0.001) when compared with the usual care group and T0 (Table 3). Compared with the sham self-acupressure and T0, the true self-acupressure outperformed significantly at both T1 (*B* = 2.25, *p* < 0.001) and T2 (*B* = 2.89, *p* < 0.001) (Table 2). At T1 and T2, between-group comparisons also found significant differences in the HADS-D scores among study groups (all *p* < 0.01), see Table 2 and Fig. 2. The true self-acupressure group showed a large effect compared with usual care at T1 (− 1.44) and a stronger effect at T2 (− 1.90), while effect sizes between the sham self-acupressure group and the usual care group at T1 and T2 were − 0.69 and − 0.86. Furthermore, the effective sizes between true self-acupressure and sham self-acupressure groups at T1 and T2 were −0.75 and −1.05 (Table 2).

### Effects of self-acupressure on quality of life

The adjusted GEE model showed that the true self-acupressure group had significant improvements in overall QoL (assessed by the FACT-B). Specifically, the FACT-B total and all subscale scores were significantly higher at T1 (all *p* < 0.05) and T2 (all *p* < 0.001) than the usual care and T0 (Table 4).

**Table 4:** Adjusted GEE model for FACT-B and subscales scores (using a first-order autoregressive correlation structure)

Predictor term	Coefficient estimate	Standard error	Wald statistic	*p*-value
Dependent variable: FACT-PWB				
Intercept	19.31	0.60	1032.90	** < 0.0001**
Group = sham SA (vs true SA)	0.43	0.93	0.21	0.6462
Group = usual care (vs true SA)	0.89	0.80	1.23	0.2676
Time = T1 (vs T0)	3.72	0.50	55.21	** < 0.0001**
Time = T2 (vs T0)	4.53	0.51	78.85	** < 0.0001**
Interaction: sham SA × T1	−2.06	0.71	8.45	**0.0036**
Interaction: usual care × T1	−3.67	0.79	21.69	** < 0.0001**
Interaction: sham SA × T2	−2.68	0.75	12.71	**0.0004**
Interaction: usual care × T2	−6.03	0.74	66.14	** < 0.0001**
Dependent variable: FACT-SWB				
Intercept	13.12	1.02	165.01	** < 0.0001**
Group = sham SA (vs true SA)	0.56	1.32	0.18	0.6691
Group = usual care (vs true SA)	0.47	1.47	0.10	0.7468
Time = T1 (vs T0)	1.64	0.79	4.32	**0.0378**
Time = T2 (vs T0)	3.44	0.92	13.87	**0.0002**
Interaction: sham SA × T1	−0.86	0.97	0.79	0.3738
Interaction: usual care × T1	−2.03	1.02	3.95	**0.0468**
Interaction: sham SA × T2	−2.63	1.08	5.90	**0.0151**
Interaction: usual care × T2	−3.93	1.13	12.14	**0.0005**
Dependent variable: FACT-EWB				
Intercept	15.78	1.36	135.49	** < 0.0001**
Group = sham SA (vs true SA)	0.02	0.98	0.00	0.9875
Group = usual care (vs true SA)	0.20	0.88	0.05	0.8227
Time = T1 (vs T0)	3.72	0.66	31.77	** < 0.0001**
Time = T2 (vs T0)	4.69	0.64	53.72	** < 0.0001**
Medical payment = mostly self-paid (> 50%)	−2.34	1.08	4.69	0.0304
Medical payment = partially self-paid (< 50%)	−0.17	1.18	0.02	0.8872
Allergy history = yes	1.94	1.07	3.32	0.0683
Primary caregiver = family member	−1.61	0.73	4.93	**0.0264**
Primary caregiver = other	−1.20	1.35	0.80	0.3721
Interaction: sham SA × T1	−1.44	0.91	2.55	0.1106
Interaction: usual care × T1	−4.28	0.89	23.16	** < 0.0001**
Interaction: sham SA × T2	−2.75	0.87	9.94	**0.0016**
Interaction: usual care × T2	−6.33	0.86	53.71	** < 0.0001**
Dependent variable: FACT-FWB				
Intercept	8.81	0.67	170.42	** < 0.0001**
Group = sham SA (vs true SA)	1.42	1.01	1.95	0.1623
Group = usual care (vs true SA)	1.92	0.99	3.74	0.0532
Time = T1 (vs T0)	4.33	0.59	53.14	** < 0.0001**
Time = T2 (vs T0)	6.00	0.71	72.00	** < 0.0001**
Interaction: sham SA × T1	−2.36	0.90	6.86	**0.0088**
Interaction: usual care × T1	−5.39	0.81	44.21	** < 0.0001**
Interaction: sham SA × T2	−3.97	0.94	17.86	** < 0.0001**
Interaction: usual care × T2	−7.64	0.91	70.17	** < 0.0001**
Dependent variable: FACT-BCS				
Intercept	23.64	0.80	878.56	** < 0.0001**
Group = sham SA (vs true SA)	0.89	1.03	0.75	0.3867
Group = usual care (vs true SA)	0.24	1.01	0.06	0.8114
Time = T1 (vs T0)	4.75	0.71	44.80	** < 0.0001**
Time = T2 (vs T0)	6.17	0.74	68.93	** < 0.0001**
Interaction: sham SA × T1	−2.92	0.95	9.38	**0.0022**
Interaction: usual care × T1	−4.06	0.94	18.57	** < 0.0001**
Interaction: sham SA × T2	−3.89	0.97	16.04	**0.0001**
Interaction: usual care × T2	−6.51	0.98	44.48	** < 0.0001**
Dependent variable: FACT total score				
Intercept	78.29	2.69	846.81	** < 0.0001**
Group = sham SA (vs true SA)	3.72	3.69	1.02	0.3136
Group = usual care (vs true SA)	3.92	3.74	1.10	0.2945
Time = T1 (vs T0)	18.17	2.27	64.23	** < 0.0001**
Time = T2 (vs T0)	24.83	2.62	90.01	** < 0.0001**
Interaction: sham SA × T1	−9.65	2.95	10.71	**0.0011**
Interaction: usual care × T1	−19.44	2.88	45.52	** < 0.0001**
Interaction: sham SA × T2	−15.92	3.20	24.69	** < 0.0001**
Interaction: usual care × T2	−30.45	3.16	93.07	** < 0.0001**

*T0* baseline, *T1* post-intervention, *T2* post-follow-up, *SA* self-acupressure, *GEE* generalised estimating equations, *FACT-B* the functional assessment of cancer therapy – breast, *PWB* physical well-being, *SWB* social well-being, *EWB* emotional well-being, *FWB* functional well-being, *BCS* breast cancer subscale, Bold entries indicate statistical significance at *p* < 0.05

Significant group-by-time interaction effects were observed for the FACT-B total score and the PWB, FWB, and BCS subscales at both T1 and T2 (all *p* < 0.05), indicating that changes over time differed across the groups (Table 4). The pattern of group-by-time interactions for the FACT-B subscales varied according to subscale, comparator and assessment point; detailed estimates and *p* values are presented in Table 4. Between-group comparisons at T1 and T2 showed that FACT-B total scores were significantly higher in both the true and sham self-acupressure groups compared with the usual care group (all *p* < 0.05); see Table 2 and Fig. 2. The difference in FACT-B total score between the true and sham self-acupressure groups was not statistically significant at T1 (*p* = 0.069) but was statistically significant at T2 (*p* = 0.0002). A confounding effect was observed with “primary caregiver (family member)” in the model (*p* = 0.026) (Table 4). At T1, true self-acupressure showed a large advantage over usual care (effect size = 0.98) and a moderate, though nonsignificant, trend compared with sham (effect size = 0.38). By T2, effects were more pronounced: true self-acupressure exceeded sham (effect size = 0.77) and usual care (effect size = 1.68). Sham also outperformed usual care (effect size = 0.91). These results confirm meaningful, sustained QoL benefits (Table 2).

### Safety of the self-acupressure

No adverse event was reported in this study.

### Costs within trial

During the trial, only one participant in each of the true self-acupressure and usual care groups reported healthcare resource use (medications plus clinical visits for symptom relief), with a sum cost of ¥931.06 and ¥2247.44, respectively. The one-off cost of video production for the true self-acupressure group was ¥2879. Given the very limited number of participants reporting resource use, the cost-effectiveness of self-acupressure intervention is not conclusive.

## Discussion

This study is a fully powered RCT to provide evidence, to the best of our knowledge, on the sustained effects of self-acupressure in managing the FSDSC and QoL in BC survivors. The results revealed statistically significant reductions in the FSDSC and improvements in QoL. These results highlight the benefits of incorporating self-acupressure into existing self-management programmes for BC survivorship care.

The encouraging results from the phase II study provided a foundation for this current full-scale trial. With a larger sample size and improved follow-up strategies, this current study confirmed and strengthened the previously observed beneficial effects of self-acupressure on FSDSC management among BC survivors and demonstrated that these effects were sustained over time through 12 weeks’ follow-up. The beneficial effects observed on individual symptoms in this study align with recent evidence confirming the effectiveness of acupressure intervention in improving the cancer-related fatigue, depressive distress and sleep issues [22, 46, 47]. This study also demonstrated significant positive effects at a cluster level, and the effect sizes of self-acupressure on the cluster-level were stronger than single symptom level. Due to the limited studies on this specific topic, direct comparisons to studies with the same participants and outcomes are not possible. However, our study findings are comparable to those from similar studies of applying self-acupressure in other types of cancer survivors and with those using other non-pharmacological interventions targeting BC survivors. For example, a pilot RCT with sham-control (38 participants in each group) examined the effects of self-acupressure and reported positive outcomes in insomnia-depression-anxiety symptom cluster from cancer survivors [40]. Although another study targeting BC survivors demonstrated the preliminary positive effects of complex multimodal intervention in alleviating the FSDSC, it required substantial professional involvement [48]. Self-acupressure in our study produced comparable benefits while being simpler and may offer a more accessible and practical alternative for symptom management for BC survivors. By addressing the symptoms of fatigue, sleep disturbance, and depression as an integrated cluster, self-acupressure showed beneficial effects at both the cluster level and on individual level. These findings also suggest potential influences on interrelationships among the three symptoms, although the underlying mechanisms require further investigation. The results of this study may imply that a cluster-based management approach could provide comprehensive and sustained benefits for cancer survivors compared with interventions targeting single symptoms alone.

This study demonstrated significant improvements in QoL, with the true self-acupressure group showing greater improvement than the other groups. This finding is consistent with previous study showing that self-acupressure is an effective method to improve QoL among BC survivors [32, 49]. Given the confirmed association between the FSDSC and QoL in the phase II RCT [27], the positive effect of self-acupressure on QoL may be attributed to improvements in physical, emotional, and functional well-being related to reduced fatigue, sleep disturbance, and depression. However, no significant between-group difference was observed in social well-being between the true self-acupressure and usual care groups after the intervention. A possible explanation is that Chinese culture places a strong emphasis on family bonds, with family members often serving as the main source of social support [50]. Previous study found that patients in China commonly seek support from family members and peer patients [51]. Therefore, participants in the intervention and control groups may have received similar levels of family support after diagnosis which may further contribute to the limited impact of the intervention on social well-being.

Consistent with the previous pilot study [27], the study results indicated that both true self-acupressure and sham self-acupressure were superior to usual care in alleviating the FSDSC at both the cluster and individual symptom levels. Of which, the true self-acupressure group was more effective in alleviating the FSDSC than other groups. Moreover, the sham self-acupressure group also outperformed the usual care group in managing the FSDSC. This finding can be explained by the potential positive changes from psychosocial factors, including participants’ strong desire for symptom relief or participants’ expectations of the acupressure intervention or active self-engagement in modulating symptom perception [52]. The reduction in the FSDSC in this current study may not be entirely attributed to specific therapeutic effects of self-acupressure but could also involve placebo effects. Even when applied to non-acupoints, it may trigger beneficial psychological, physiological, and behavioural responses [29, 53]. Such placebo effects highlight the importance of using these psychological factors in designing symptom management interventions for BC survivors. The findings regarding the placebo effects of acupressure are consistent with a previous systematic review on sham acupressure [29].

Medical payment showed a significant association with fatigue and the FSDSC in the adjusted GEE model. Specifically, partial self-payment (less than 50%) of medical payment was associated with a lower FSDSC composite score (*p* = 0.037). Such findings indicated that financial burden may contribute to symptom burden in BC survivors. However, self-acupressure remained significantly effective after accounting for these covariates. Financial burden may increase psychological distress, limit access to supportive care resources, and reduce adherence to self-management activities, thereby exacerbating the overall symptom experience [54, 55]. Therefore, future studies could consider incorporating stratified randomisation based on medical payment type to achieve better group balance and improve internal validity.

In terms of the cost-effectiveness of self-acupressure in this study, making robust conclusions is challenging due to the limited data within the trial. It is also possible some over-the-counter medicines may have been used but not captured as not all information on medicine use was available in the medical records. In addition, relying on participants’ self-report and recall may have led to incomplete data. A more comprehensive economic evaluation, incorporating costs from the healthcare provider perspective, the societal perspective, and the patient perspective, is warranted to further assess the value of acupressure in both clinical practice and future trials [56].

## Implications

Findings from this phase III RCT, together with previous related studies [26–28], supported that self-acupressure is a feasible, safe, and effective intervention for symptom management in BC survivors. Self-acupressure requires minimal pre-training and can be performed independently at any time in any setting once the technique has been learned. Such features of self-acupressure make it highly appropriate for primary healthcare where accessible, self-managed, low-burden strategies are essential. Moreover, the evidence-based self-acupressure intervention protocol [26] provides a standardised approach that can be translated into routine practice to support symptom management for BC survivors. Although participants generally adhered to the daily 36-min practice in this study, sustaining continuous practice for the full 49 days was challenging. In real-world implementation, individualised adjustments in sessions could be made based on participants’ functional status, symptom burden, and personal preferences, thereby supporting adherence to the overall intervention regimen.

## Limitations

This study has several limitations. The assessment of clinical outcomes relied on participants’ self-reported measures, which could be quite subjective sometimes despite the fact that the measures used in this study are well-validated. Future studies should include objective measures, such as biomarkers or actigraphy, to supplement self-reported assessments of symptoms. Due to the lack of a validated instrument for assessing the FSDSC at the cluster level, the composite score was calculated as the average of three separate NRS ratings. Despite this method was used in previous studies [39, 40], this approach implicitly assumes equal weighting of each symptom and may not reflect potential interactions among symptoms. While potential mechanisms of self-acupressure (e.g. decreased pro-inflammatory cytokines) can be theoretically substantiated, these cytokines were not directly assessed within the scope of the study.

## Conclusion

This clinical trial was theory-driven and practice-based, following an evidence-based intervention protocol with promising findings and subsequent protocol refinements. It provides robust evidence on the positive effects of self-acupressure in improving the FSDSC and QoL in BC survivors. Self-acupressure could be safely integrated into clinical practice for BC survivors with symptom burden.

## Supplementary information

Below is the link to the electronic supplementary material.

ESM 1 (DOCX 1.19 MB)

## Data Availability

Individual participant data are not available for sharing in accordance with the ethical requirements of the study.
