# Supplementary material for: Effects of self-acupressure on the fatigue-sleep disturbance-depression symptom cluster in breast cancer survivors: a phase III randomised controlled trial
Source: Support Care Cancer. 2026 Sep 17;34(10):981. doi: 10.1007/s00520-026-11155-2 (PMC13582098; doi:10.1007/s00520-026-11155-2)
Supplement: Supplementary file 1 [file 520_2026_11155_MOESM1_ESM.docx]

**Illustration of acupoint locations for true self-acupressure group (In Chinese)**

**Illustration of acupoint locations for sham self-acupressure group (In Chinese)**
